# (*E*,*E*)-1,5-Di-2-thienylpenta-1,4-dien-3-one

**DOI:** 10.1107/S1600536808026603

**Published:** 2008-08-23

**Authors:** S. Murugavel, G. Ganesh, A. SubbiahPandi, Ramalingam Murugan, S. SrimanNarayanan

**Affiliations:** a Department of Physics, Thanthai Periyar Government Institute of Technology, Vellore 632 002, India; b Department of Physics, SMK Fomra Institute of Technology, Thaiyur, Chennai 603 103, India; c Department of Physics, Presidency College (Autonomous), Chennai 600 005, India; d Department of Analytical Chemistry, University of Madras, Guindy Campus, Chennai 600 025, India

## Abstract

In the title compound, C_13_H_10_OS_2_, the dihedral angle between the thio­phene rings is 14.3 (1)°. The mol­ecular structure is stabilized by C—H⋯π inter­actions between a thio­phene H atom and an adjacent thio­phene ring, and by inter­molecular C—H⋯O hydrogen bonds.

## Related literature

For the bioactivity of chalcones, see: Go *et al.* (2005 ▶). For uses in organic solid-state photochemistry, see: Gould *et al.* (1995 ▶); For a related structure, see: Arshad *et al.* (2008 ▶). For the non-linear optical properties of bis-chalcones, see: Uchida *et al.* (1998 ▶).
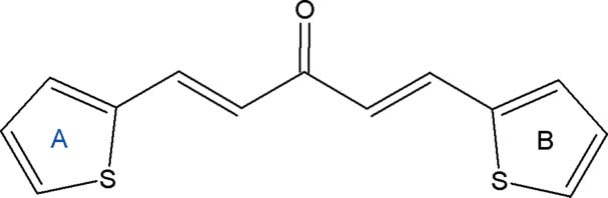


## Experimental

### 

#### Crystal data

C_13_H_10_OS_2_
*M* *_r_* = 246.35
Orthorhombic, 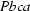

*a* = 12.1141 (4) Å
*b* = 7.4449 (3) Å
*c* = 27.246 (1) Å
*V* = 2457.27 (16) Å^3^
*Z* = 8
Mo *K*α radiation
μ = 0.41 mm^−1^
*T* = 293 (2) K
0.26 × 0.15 × 0.15 mm

#### Data collection

Bruker APEXII CCD area-detector diffractometer
Absorption correction: multi-scan (*SADABS*; Sheldrick, 1996 ▶) *T*_min_ = 0.984, *T*_max_ = 0.987
13976 measured reflections
2373 independent reflections
1719 reflections with *I* > 2σ(*I*)
*R*_int_ = 0.023

#### Refinement

*R*[*F* ^2^ > 2σ(*F* ^2^)] = 0.057
*wR*(*F* ^2^) = 0.210
*S* = 1.01
2373 reflections
145 parameters
H-atom parameters constrained
Δρ_max_ = 0.49 e Å^−3^
Δρ_min_ = −0.36 e Å^−3^

### 

Data collection: *APEX2* (Bruker, 2004 ▶); cell refinement: *APEX2* and *SAINT* (Bruker, 2004 ▶); data reduction: *SAINT* and *XPREP* (Bruker, 2004 ▶); program(s) used to solve structure: *SHELXS97* (Sheldrick, 2008 ▶); program(s) used to refine structure: *SHELXL97* (Sheldrick, 2008 ▶); molecular graphics: *ORTEP-3* (Farrugia (1997 ▶); software used to prepare material for publication: *SHELXL97* and *PLATON* (Spek, 2003 ▶).

## Supplementary Material

Crystal structure: contains datablocks global, I. DOI: 10.1107/S1600536808026603/lx2066sup1.cif

Structure factors: contains datablocks I. DOI: 10.1107/S1600536808026603/lx2066Isup2.hkl

Additional supplementary materials:  crystallographic information; 3D view; checkCIF report

## Figures and Tables

**Table 1:** Hydrogen-bond geometry (Å, °)

*D*—H⋯*A*	*D*—H	H⋯*A*	*D*⋯*A*	*D*—H⋯*A*
C1—H1⋯O1^i^	0.93	2.47	3.374 (5)	165
C13—H13⋯O1^ii^	0.93	2.33	3.255 (4)	171
C11—H11⋯*Cg*^iii^	0.93	3.12	3.936 (5)	148

Symmetry codes: (i) 
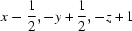
; (ii) 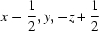; (iii) 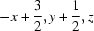. *Cg* is the centroid of the C10/C11/C12/C13/S1 thio­phene ring.

**Fig. 1.**
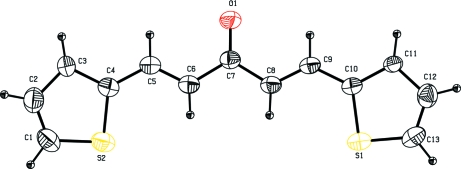
The molecular structure of title compound showing 30% probability displacement ellipsoids.

**Fig. 2.**
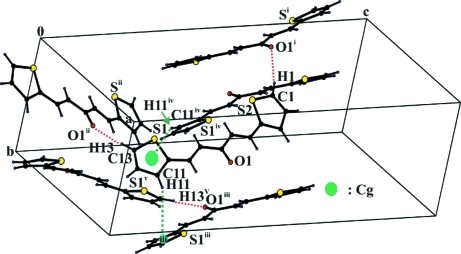
C—H···π and C—H···O interactions (dotted lines) in the title compound. Cg denotes the ring centroid. [Symmetry code: (i) x-1/2, -y+1/2, -z+1; (ii)x-1/2, y, -z+1/2; (iii) x-1/2, y, -z+1/2; (iv) -x+3/2, y-1/2, z; (v)-x+1, y+1/2, -z+1/2.]

## supplementary crystallographic information

### Comment

Chalcones with the general formula Ar—CH═CH—CO—Ar are an important class of compounds, with the common structural entity being the central –CH═CH—C(═O)- group, in the H atoms can be substituted. The –C═C– double bond can be photoreactive and can produce various products through solid-state photo cycloaddition. Therefore, chalcones are widely used in organic solid-state photochemistry (Gould *et al.*, 1995). Reviews on the bioactivities of various chalcones have been reported (Go *et al.*, 2005). Bis-chalcones are also found to exhibit good NLO properties (Uchida *et al.*, 1998). In view of this biological importance, the crystal structure of the title compound (I), (1E, 4E)-1,5-Bis(2-thienyl)penta-1,4-dien-3-one (Fig. 1) has been determined and the results are presented here.

Compound (I) consists of two thiophene rings A and B. The central position of (I) shows double and single bonds orientating from O1 atom and five C atom behave like a backbone. The planarities of rings A and B are fairly good. The bond lengths in the (I) are normal and comparable with the corresponding values observed in the related structure (Arshad *et al.*, 2008). The dihedral angle between the two thiophene rings is 14.3 (1)°. The crystal packing (Fig. 2) is stabilized by C—H···π interactions between a thiophene H atom and a neighbouring thiophene ring, with a C11—H11···Cg^iii^ separetion of 2.34 Å (Fig. 2 and Table 1; Cg is the centroid of the C10/C11/C12/C13/S1 thiophene ring, symmetry code as in Fig. 2). The molecular packing is further stabilized by intermolecular C—H···O hydrogen bonds (Fig. 2 and Table 1; symmetry code as in Fig. 2).

### Experimental

A solution of sodium hydroxide (10 g, 0.25 mol) in water (50 ml) was added to a solution of acetone (5 ml) and 2-thiophenecarboxaldehyde (22.4 g, 0.2 mol) in methanol (50 ml) at 273 K. This mixture was stirred overnight and the product was filtered. Single crystals suitable for X-ray diffraction was obtained by slow evaporation of a solution of the title compound in ethyl acetate.

### Refinement

All H atoms were fixed geometrically and allowed to ride on their parent atoms, with N—H=0.86Å and C—H= 0.93–0.98Å with *U*_iso_(H)= 1.5*U*_eq_(methyl H) and 1.2*U*_eq_(for other H atoms).

### Crystal data

**Table:**

C_13_H_10_OS_2_	*F*_000_ = 1024
*M**_r_* = 246.35	*D*_x_ = 1.332 Mg m^−^^3^
Orthorhombic, *P**b**c**a*	Mo *K*α radiation λ = 0.71073 Å
Hall symbol: -P 2ac 2ab	Cell parameters from 10259 reflections
*a* = 12.1141 (4) Å	θ = 2.3–30.3º
*b* = 7.4449 (3) Å	µ = 0.41 mm^−^^1^
*c* = 27.246 (1) Å	*T* = 293 (2) K
*V* = 2457.27 (16) Å^3^	Block, colourless
*Z* = 8	0.26 × 0.15 × 0.15 mm

### Data collection

**Table:**

Bruker APEXII CCD area-detector diffractometer	2373 independent reflections
Radiation source: fine-focus sealed tube	1719 reflections with *I* > 2σ(*I*)
Monochromator: graphite	*R*_int_ = 0.023
Detector resolution: 10 pixels mm^-1^	θ_max_ = 26.0º
*T* = 293(2) K	θ_min_ = 1.5º
ω scans	*h* = −11→14
Absorption correction: Multi-scan(SADABS; Sheldrick, 1996)	*k* = −7→9
*T*_min_ = 0.984, *T*_max_ = 0.987	*l* = −32→31
13976 measured reflections	

### Refinement

**Table:**

Refinement on *F*^2^	Secondary atom site location: difference Fourier map
Least-squares matrix: full	Hydrogen site location: inferred from neighbouring sites
*R*[*F*^2^ > 2σ(*F*^2^)] = 0.057	H-atom parameters constrained
*wR*(*F*^2^) = 0.211	*w* = 1/[σ^2^(*F*_o_^2^) + (0.1295*P*)^2^ + 1.4091*P*] where *P* = (*F*_o_^2^ + 2*F*_c_^2^)/3
*S* = 1.01	(Δ/σ)_max_ < 0.001
2373 reflections	Δρ_max_ = 0.49 e Å^−^^3^
145 parameters	Δρ_min_ = −0.36 e Å^−^^3^
Primary atom site location: structure-invariant direct methods	Extinction correction: none

### Special details

**Table:**

Geometry. All esds (except the esd in the dihedral angle between two l.s. planes) are estimated using the full covariance matrix. The cell esds are taken into account individually in the estimation of esds in distances, angles and torsion angles; correlations between esds in cell parameters are only used when they are defined by crystal symmetry. An approximate (isotropic) treatment of cell esds is used for estimating esds involving l.s. planes.
Refinement. Refinement of F^2^ against ALL reflections. The weighted R-factor wR and goodness of fit S are based on F^2^, conventional R-factors R are based on F, with F set to zero for negative F^2^. The threshold expression of F^2^ > 2sigma(F^2^) is used only for calculating R-factors(gt) etc. and is not relevant to the choice of reflections for refinement. R-factors based on F^2^ are statistically about twice as large as those based on F, and R- factors based on ALL data will be even larger.

### Fractional atomic coordinates and isotropic or equivalent isotropic displacement parameters (Å^2^)

**Table:**

	*x*	*y*	*z*	*U*_iso_*/*U*_eq_	
S1	0.52710 (7)	0.54328 (13)	0.23889 (4)	0.0733 (4)	
S2	0.53529 (8)	0.23805 (14)	0.51370 (4)	0.0801 (4)	
O1	0.82061 (18)	0.5548 (4)	0.38920 (9)	0.0734 (7)	
C1	0.5312 (3)	0.2113 (6)	0.57495 (18)	0.0876 (13)	
H1	0.4768	0.1455	0.5910	0.105*	
C2	0.6135 (4)	0.2934 (6)	0.59817 (16)	0.0907 (12)	
H2	0.6209	0.2904	0.6321	0.109*	
C3	0.6903 (3)	0.3874 (5)	0.56716 (12)	0.0667 (9)	
H3	0.7525	0.4507	0.5773	0.080*	
C4	0.6519 (3)	0.3642 (4)	0.51665 (13)	0.0612 (8)	
C5	0.7055 (3)	0.4350 (4)	0.47339 (13)	0.0610 (8)	
H5	0.7755	0.4843	0.4774	0.073*	
C6	0.6638 (3)	0.4359 (4)	0.42854 (13)	0.0612 (8)	
H6	0.5938	0.3881	0.4236	0.073*	
C7	0.7236 (2)	0.5093 (4)	0.38617 (12)	0.0573 (8)	
C8	0.6611 (2)	0.5283 (4)	0.34019 (12)	0.0594 (8)	
H8	0.5901	0.4810	0.3384	0.071*	
C9	0.7027 (2)	0.6109 (4)	0.30106 (12)	0.0565 (7)	
H9	0.7733	0.6583	0.3045	0.068*	
C10	0.6513 (2)	0.6354 (4)	0.25413 (12)	0.0536 (7)	
C11	0.6974 (2)	0.7299 (4)	0.21337 (11)	0.0541 (7)	
H11	0.7653	0.7882	0.2132	0.065*	
C12	0.6228 (3)	0.7207 (6)	0.17301 (14)	0.0794 (10)	
H12	0.6364	0.7764	0.1431	0.095*	
C13	0.5322 (3)	0.6260 (6)	0.18183 (15)	0.0774 (11)	
H13	0.4771	0.6074	0.1586	0.093*	

### Atomic displacement parameters (Å^2^)

**Table:**

	*U*^11^	*U*^22^	*U*^33^	*U*^12^	*U*^13^	*U*^23^
S1	0.0542 (6)	0.0750 (6)	0.0909 (8)	−0.0090 (4)	−0.0089 (4)	−0.0068 (5)
S2	0.0619 (6)	0.0821 (7)	0.0962 (9)	−0.0065 (4)	0.0047 (4)	0.0076 (5)
O1	0.0483 (13)	0.1008 (18)	0.0710 (16)	−0.0067 (11)	0.0009 (10)	−0.0029 (12)
C1	0.069 (2)	0.090 (3)	0.103 (3)	0.0109 (19)	0.018 (2)	0.033 (2)
C2	0.091 (3)	0.113 (3)	0.068 (2)	0.012 (3)	0.000 (2)	0.013 (2)
C3	0.071 (2)	0.073 (2)	0.056 (2)	−0.0057 (16)	−0.0001 (15)	0.0110 (15)
C4	0.0558 (17)	0.0542 (15)	0.074 (2)	0.0020 (13)	0.0024 (14)	0.0003 (14)
C5	0.0527 (16)	0.0593 (17)	0.071 (2)	−0.0027 (13)	−0.0005 (15)	−0.0028 (14)
C6	0.0510 (16)	0.0621 (17)	0.071 (2)	−0.0018 (13)	0.0012 (14)	−0.0036 (15)
C7	0.0497 (17)	0.0576 (16)	0.065 (2)	0.0021 (13)	0.0033 (13)	−0.0081 (14)
C8	0.0467 (15)	0.0646 (17)	0.067 (2)	−0.0029 (13)	0.0012 (14)	−0.0081 (15)
C9	0.0435 (14)	0.0592 (16)	0.067 (2)	−0.0007 (12)	−0.0031 (13)	−0.0097 (14)
C10	0.0457 (15)	0.0511 (14)	0.0641 (19)	0.0013 (12)	−0.0020 (13)	−0.0103 (13)
C11	0.0442 (14)	0.0624 (17)	0.0558 (18)	−0.0046 (12)	−0.0030 (12)	−0.0082 (13)
C12	0.081 (2)	0.096 (3)	0.062 (2)	0.013 (2)	0.0044 (18)	−0.0026 (18)
C13	0.063 (2)	0.091 (3)	0.078 (3)	0.0085 (18)	−0.0146 (17)	−0.022 (2)

### Geometric parameters (Å, °)

**Table:**

S1—C13	1.673 (5)	C6—C7	1.468 (5)
S1—C10	1.704 (3)	C6—H6	0.9300
S2—C1	1.682 (5)	C7—C8	1.471 (5)
S2—C4	1.699 (3)	C8—C9	1.330 (5)
O1—C7	1.225 (4)	C8—H8	0.9300
C1—C2	1.330 (6)	C9—C10	1.434 (4)
C1—H1	0.9300	C9—H9	0.9300
C2—C3	1.438 (5)	C10—C11	1.429 (4)
C2—H2	0.9300	C11—C12	1.425 (5)
C3—C4	1.463 (5)	C11—H11	0.9300
C3—H3	0.9300	C12—C13	1.326 (5)
C4—C5	1.445 (5)	C12—H12	0.9300
C5—C6	1.322 (5)	C13—H13	0.9300
C5—H5	0.9300		
			
C13—S1—C10	92.62 (17)	O1—C7—C8	121.6 (3)
C1—S2—C4	92.5 (2)	C6—C7—C8	116.8 (3)
C2—C1—S2	113.3 (3)	C9—C8—C7	122.1 (3)
C2—C1—H1	123.3	C9—C8—H8	118.9
S2—C1—H1	123.3	C7—C8—H8	118.9
C1—C2—C3	115.4 (4)	C8—C9—C10	127.5 (3)
C1—C2—H2	122.3	C8—C9—H9	116.3
C3—C2—H2	122.3	C10—C9—H9	116.3
C2—C3—C4	106.8 (3)	C9—C10—C11	125.9 (3)
C2—C3—H3	126.6	C9—C10—S1	123.3 (2)
C4—C3—H3	126.6	C11—C10—S1	110.7 (2)
C5—C4—C3	125.6 (3)	C12—C11—C10	109.1 (3)
C5—C4—S2	122.5 (3)	C12—C11—H11	125.4
C3—C4—S2	112.0 (2)	C10—C11—H11	125.4
C6—C5—C4	125.7 (3)	C13—C12—C11	114.2 (4)
C6—C5—H5	117.1	C13—C12—H12	122.9
C4—C5—H5	117.1	C11—C12—H12	122.9
C5—C6—C7	122.7 (3)	C12—C13—S1	113.3 (3)
C5—C6—H6	118.7	C12—C13—H13	123.4
C7—C6—H6	118.7	S1—C13—H13	123.4
O1—C7—C6	121.5 (3)		
			
C4—S2—C1—C2	0.3 (4)	O1—C7—C8—C9	−6.5 (5)
S2—C1—C2—C3	−0.5 (5)	C6—C7—C8—C9	172.5 (3)
C1—C2—C3—C4	0.5 (5)	C7—C8—C9—C10	178.8 (3)
C2—C3—C4—C5	−179.2 (3)	C8—C9—C10—C11	177.9 (3)
C2—C3—C4—S2	−0.3 (4)	C8—C9—C10—S1	−5.4 (4)
C1—S2—C4—C5	179.0 (3)	C13—S1—C10—C9	−177.9 (3)
C1—S2—C4—C3	0.1 (3)	C13—S1—C10—C11	−0.8 (2)
C3—C4—C5—C6	−170.2 (3)	C9—C10—C11—C12	178.5 (3)
S2—C4—C5—C6	11.1 (5)	S1—C10—C11—C12	1.5 (3)
C4—C5—C6—C7	−179.5 (3)	C10—C11—C12—C13	−1.6 (4)
C5—C6—C7—O1	8.6 (5)	C11—C12—C13—S1	1.0 (4)
C5—C6—C7—C8	−170.4 (3)	C10—S1—C13—C12	−0.1 (3)

### Hydrogen-bond geometry (Å, °)

**Table:**

*D*—H···*A*	*D*—H	H···*A*	*D*···*A*	*D*—H···*A*
C1—H1···O1^i^	0.93	2.47	3.374 (5)	165
C13—H13···O1^ii^	0.93	2.33	3.255 (4)	171
C11—H11···Cg^iii^	0.93	3.12	3.936 (5)	148

Symmetry codes: (i) *x*−1/2, −*y*+1/2, −*z*+1; (ii) *x*−1/2, *y*, −*z*+1/2; (iii) −*x*+3/2, *y*+1/2, *z*.
